# PET Foams Surface Treated with Graphene Nanoplatelets: Evaluation of Thermal Resistance and Flame Retardancy

**DOI:** 10.3390/polym13040501

**Published:** 2021-02-06

**Authors:** Samuele Matta, Laura Giorgia Rizzi, Alberto Frache

**Affiliations:** 1 Department of Applied Science and Technology, Politecnico di Torino, Alessandria Campus, Viale Teresa Michel 5, 15121 Alessandria, Italy; samuele.matta@polito.it; 2 Directa Plus S.p.A., c/o ComoNExT—Science and Technology Park, Via Cavour 2, 22074 Lomazzo, Italy; laura.rizzi@directa-plus.com; 3 INSTM Research Unit, Politecnico di Torino, Viale Teresa Michel 5, 15121 Alessandria, Italy

**Keywords:** PSS dispersant, cone calorimetry, flame penetration, flame-retardant surface coating, surface ageing

## Abstract

In this work, fire-retardant systems consisting of graphene nanoplatelets (GNPs) and dispersant agents were designed and applied on polyethylene terephthalate (PET) foam. Manual deposition from three different liquid solutions was performed in order to create a protective coating on the specimen’s surface. A very low amount of coating, between 1.5 and 3.5 wt%, was chosen for the preparation of coated samples. Flammability, flame penetration, and combustion tests demonstrated the improvement provided to the foam via coating. In particular, specimens with PSS/GNPs coating, compared to neat foam, were able to interrupt the flame during horizontal and vertical flammability tests and led to longer endurance times during the flame penetration test. Furthermore, during cone calorimetry tests, the time to ignition (TTI) increased and the peak of heat release rate (pHRR) was drastically reduced by up to 60% compared to that of the uncoated PET foam. Finally, ageing for 48 and 115 h at 160 °C was performed on coated specimens to evaluate the effect on flammability and combustion behavior. Scanning electron microscopy (SEM) images proved the morphological effect of the heat treatment on the surface, showing that the coating was uniformly distributed. In this case, fire-retardant properties were enhanced, even if fewer GNPs were used.

## 1. Introduction

The use of polymer foams is widespread in many fields and current technologies. Indeed, they can be found in various applications such as furniture, packaging, and insulator materials. Compared to the original polymer, compressibility, thermal insulation, and lightness are all enhanced in foams [1]. This combination of properties makes foams ideal materials for transportation, building insulation, packaging, cushioning, food and drink containers, furniture, gaskets, sports applications, and shock and sound attenuation [2]. Despite the numerous advantages of these materials, one of the major drawbacks is their high flammability. Their organic nature is the cause of their intrinsic fire hazard as they can easily ignite and burn fast when subjected to a heat source. In case of ignition, polymeric materials not only fuel the fire, but also could release dangerous gases and smoke [3]. Consequently, improving the fire-retardant behavior of polymers is a major challenge for extending their use in most applications.

The polymer surface is the critical zone for flammability during a combustion scenario because, as the interface between the gas and condensed phase, it controls mass and heat transfers, which are the processes responsible for fueling the flames. Indeed, heat reaching the polymer surface is transmitted to the polymer bulk, from which volatile products of thermal decomposition diffuse towards the surface and the gas phase, feeding the flame. The polymer surface thus plays a key role in polymer ignition and combustion because it has chemical and physical characteristics that affect the flux of the combustible volatiles towards the gas phase. Indeed, the production or accumulation of a thermally stable surface coating, which acts as a barrier to mass and heat exchange, is one of the most used fire-retardant strategies performed via bulk addition [4]. The bulk addition of fire-retardant additives (metal hydroxides) could require up to 60% loading, changing other properties of the materials such as their mechanical properties and density. Polymer nanocomposites are a class of materials in which the filler phase reaches the nanometer scale, so at least one dimension of the filler phase is less than 100 nm [5]. Polymer nanocomposites show considerable improvement of properties at much lower loadings than polymer composites with common microscale fillers [6]. In a previous study (Trusiano et al., 2019 [7]), it was shown that the preparation of polypropylene-based nanocomposites exhibits better thermal stability and a lower combustion rate starting from the use of just 1 wt% of graphene nanoplatelets as filler. The thermo-oxidative analysis presents higher temperatures of starting degradation for graphene nanoplatelet (GNP) nanocomposites (245 and 250 °C, for 1 and 3 wt% GNPs, respectively) in comparison to pure polymer (235 °C). The results of cone calorimeter tests show a decrease in the peak of the heat release rate (pHRR) of nanocomposites with respect to pure polymer, from 1218 kW m^−2^ of PP to 1001 and 907 kW m^−2^ for 1 and 3 wt% GNPs, respectively, using a heat flux of 35 kW m^−2^. After increasing the heat flux of the instrument to 50 kW m^−2^, the pHRR decreases to 37% for the 3% GNP-nanocomposites, compared to the neat polymer. The effect of combustion on polymer composites with bulk dispersion of the filler has been deeply studied and the literature is full of examples. For polymer-layered silicate nanocomposites, a change in the structure of the materials was observed during heating. Indeed, the filler can migrate to and accumulate on the surface of the polymer due to heating before the combustion. This effect permits the creation of a protective barrier that is beneficial for the flame-retardant properties of the material [8,9]. This behavior was also investigated by Pastore et al. [10] on nanocomposites based on ethylene vinyl acetate (EVA) intercalated with modified clay. Indeed, using in situ high-temperature X-ray diffraction (HT-XRD), rearrangement of the nanocomposite morphology between 75 and 350 °C was demonstrated in an oxidizing environment, leading to exfoliation. Colonna et al. studied the effect of the migration of layered silicate on the fire-retardant properties and ageing of the material using temperatures near the melting point. Ageing promotes the formation of a barrier that strongly affects the combustion behavior compared to unaged material. EVA—organically modified clay (Cloisite 20A)—nanocomposites were prepared and cone calorimeter tests showed that the aged material presents a time to ignition (TTI) of 110 s, which is an important achievement compared to the 65 s of pure polymer and 64 s of the unaged sample. With respect to pure polymer, aged material exhibits a sharp decrease in pHRR (from 1380 to 657 kW m^−2^), but this is higher than that of unaged nanocomposites (430 kW m^−2^) [11].

An alternative method for preparing fire-retardant composites is based on providing the polymeric material with a superficial coating to protect the bulk from heat and delay the production of volatiles while maintaining the properties of the bulk material. Several strategies of modification of the polymer surface exist in the literature, and the most used are layer-by-layer (LbL) deposition, sol-gel treatments, and the use of intumescent coating. LbL assembly is a powerful strategy for controlling the deposition of coatings on a surface, exploiting the interactions between selected reagents for the adsorption on a substrate. Nowadays, the electrostatic attraction between positively and negatively charged solutions/suspensions of polyelectrolytes or nanoparticles is the most frequently used strategy for LBL deposition [4,12]. One of the applications of LbL is for the deposition of flame-retardant protection coatings. For example, Carosio et al. showed for the first time an LbL deposition treatment on closed-cell polyethylene terephthalate (PET) foam with films of ammonium polyphosphate (APP) and deoxyribonucleic acid (DNA) for the application of flame retardant. The superior performances of APP coatings are able to suppress the melt dripping behavior during flammability tests and reduce the pHRR by 25% during cone calorimetry tests [13].

Sol-gel techniques for obtaining silica-nanoparticles have been widely studied in the field of polymeric materials in order to reduce the flammability of many classes of polymers (epoxy [14,15] and phenolic resins [16], polymethylmethacrylates [17], polyesters [18]) and textiles [19]. In the work of Alongi et al., PET, cotton, and relative blends of textile fabrics were treated via sol-gel processes using tetraethoxysilane (TEOS) as an inorganic precursor. The presence of silica film was responsible for the improvement in thermal stability and strongly affected the combustion behavior. In this case, the sol-gel process could be effectively used to improve the flame retardancy of cotton-based textiles but did not improve the fire retardancy of PET [20].

Organic intumescent coatings are typically based on a mixture of an inorganic acid or its thermal precursor such as ammonium polyphosphate (APP), a source of carbon such as a polyol, and a “spumific” compound such as melamine that is able to release gases to foam the charring coating and a binder resin [4]. S. Bourbigot et al. studied different types of commercial intumescent coatings applied to glass fiber epoxy/PET foam sandwich composites and tested them in fire tunnels, demonstrating a significant reduction of the substrate temperature, which is clearly correlated with the fire resistance of the composite panels [21].

The present study shows a simple and fast method for creating an innovative flame-retardant coating on PET foam using graphene nanoplatelets (GNPs) via a water solution. The aim is to investigate the flammability, combustion properties, and morphology of the coated samples. In addition, it is demonstrated that the ageing of the samples causes rearrangements of the morphology of GNPs on the surface, leading to a uniformly distributed coating. The consequential benefits in terms of flame-retardant behavior are studied. Therefore, it is possible to obtain a material with building applications subjected to a single post-treatment directly on the finished object (avoiding compounding operations) with relevant antiflame performance using a limited quantity of charge.

## 2. Materials and Methods

### 2.1. Materials

Closed-cell polyethylene terephthalate foams with a density of 120 kg/m^3^ were produced by BASF (Ludwigshafen, Germany). Liquid G+ (the commercial name for highly concentrated water-based graphene nanoplatelet dispersions) was produced by Directa Plus (Lomazzo, Italy). The production process includes the following steps: preparation of expanded graphite from commercially available expandable graphite (grade ES 250 F5 from Graphit Kropfmühl AG, Hauzenberg, Germany); carbon content, minimum 90%; expansion, 250 cm^3^; start temperature, 200–230 °C; particle size, 80% >300 µm); mixing of surfactant and expanded graphite in a cooled, stirred tank; and subjecting the mixture to ultrasound treatment with an industrial ultrasonicator (UIP2000hdT unit from Hielscher Ultrasonics GmbH, Teltow, Germany) to exfoliate the graphene nanoplatelets (GNPs). The expanded graphite is obtained by processing the expandable graphite through a proprietary plasma torch, where the resulting expanded graphite has an apparent density of about 2.5 g/L. The ultrasound treatment is administered through an ultrasound flow cell at full amplitude (about 80 µm), with a working ultrasound power of about 1500 W and treatment duration of about 14 h per liter of dispersion. The surfactant is added to the dispersion at a concentration of 10% by the weight of graphite. This physical–mechanical exfoliation method permits the liquid dispersion of GNPs at a concentration of 200 g/L in a water solution, explained in detail in a patent [22]. The resulting GNPs are characterized by a high aspect ratio (length to thickness > 1000), lateral dimensions < 1–2 µm, and thickness typically below 3 nm) [23].

**Figure 1:**
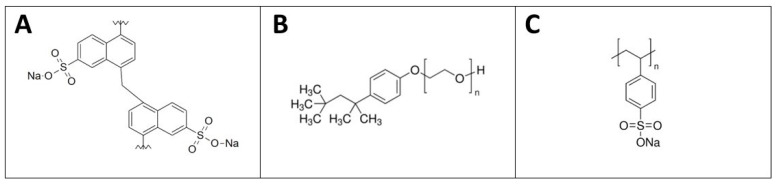
The chemical structures of poly(di-2-naftalensolphonate of sodium) (DNMS) (**A**), Triton X-100 (**B**), and poly(sodium 4-styrenesulfonate) (PSS) (**C**).

Three different types of water-based GNPs dispersions were used with the same ratio of GNPs/dispersant equal to 10:1, respectively. The ratio of 10:1 originated from an optimization phase: the minimum amount of surfactant with respect to the GNPs’ solid content which could support good dispersion—i.e., preventing agglomeration—was found. The dispersants exploited were the following: poly(di-2-naftalensolphonate of sodium) (DNMS) (Bozzetto Group—Filago, Italy), Triton X-100 (Sigma-Aldrich, St. Louis, MI, USA), poly(sodium 4-styrenesulfonate) (PSS) (Sigma-Aldrich) (Figure 1). The dynamic viscosity values of the dispersants were 278, 4077 and 185 cP, respectively.

### 2.2. Characterization Techniques

The samples’ morphologies were characterized using an LEO 1450VP (Zeiss, Oberkochen, Germany scanning electron microscope (beam voltage, 20 kV; working distance, 15 mm). Cryogenic fracture surfaces were obtained via prior immersion of the specimens in liquid nitrogen. The samples were fixed on conductive adhesive tape and gold-metallized. Flammability tests were performed both in horizontal and vertical configurations on 150 × 50 × 20 mm^3^ specimens.

The horizontal burning sample was marked at 25 mm and 125 mm from the application of the flame to evaluate the linear burning rate at 100 mm (ASTM D 635). The specimen was placed in a horizontal position on wire gauze and was ignited from the short side by a 25-mm methane flame, tilted at 45°. For coated specimens, three applications of the flame were necessary (10, 10, and 30 s, respectively) to reach the first mark and start the calculation of the burning rate. For the evaluation of incandescent melt dripping, a cotton wad was placed under the specimen. Burning time, burning rate, final residue, and melt dripping were registered.

The vertical burning tests were performed following the UL-94 Vertical procedure (ASTM D 3801). The sample was held in the vertical position by a metallic clamp and was ignited from the bottom using a 20-mm methane flame tilted at 45°. The flame was applied twice for 10 s and the afterflame times were recorded. A cotton wad was positioned at 30 cm under the bottom of the specimen to evaluate incandescent dripping. Times of afterflame burning and the evolution of fire, final residue, and melt dripping were registered.

**Figure 2:**
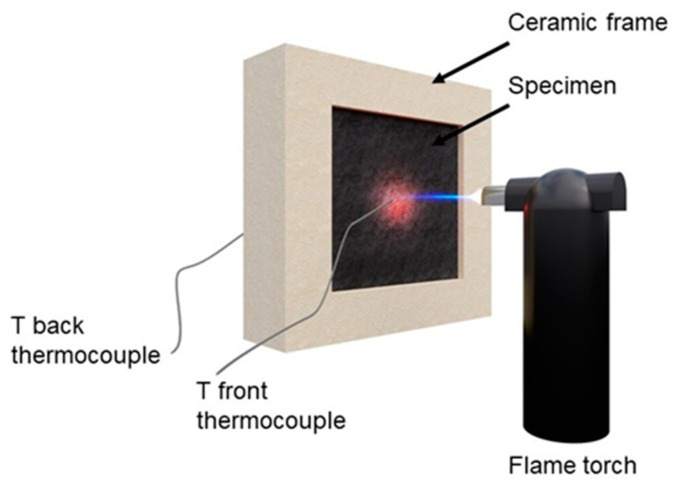
The scheme of the flame penetration system.

A flame penetration test was performed on a 40 × 40 × 2 mm^3^ sample placed inside a ceramic frame and kept in the horizontal position. One side was exposed to a butane torch with a flame pointed at the center of the specimen. The flame length was 25 mm and was held 70 mm away from the specimen’s surface. The continuous flame was applied until complete perforation of the sample from one side to other, and the time was recorded (a standard error of ±5 s was evaluated for all the tests). Two thermocouples were exploited to register the temperatures of the exposed and unexposed sides. A schematic representation of the system is reported in Figure 2.

The combustion behavior was investigated by cone calorimeter according to the ISO 5660 standard using a Fire Testing Technology (FTT, West Sussex, UK) instrument. The square samples (100 × 100 × 20 mm^3^) were exposed to a 35 kW/m^2^ heat flux in a horizontal configuration. The specimen was wrapped in aluminum foil, except the upper face, and placed on a load cell maintaining 25 mm between the surface and cone heater. The time to ignition (TTI, s), peak of heat release rate (pHRR, kW m^−2^), total heat release (THR, MJ m^−2^), and final residue mass were measured.

For flammability, flame penetration, and combustion tests, the samples were conditioned for at least 48 h in a climatic chamber with a controlled temperature and relative humidity of 23 ± 1 °C and 50%, respectively. Samples were removed just before starting the tests. Furthermore, the tests were repeated at least three times for every formulation to evaluate the reproducibility of the results. For all parameters, the average result and standard deviation are indicated.

In order to evaluate the effects of ageing on fire-retardant properties, selected formulations were heat-treated at 160 °C. Annealing took place in an oven for 48 or 115 h.

### 2.3. Liquid G+ Deposition Procedure

The samples were obtained from the PET foam plate with the dimensions required by the tests. Then, the liquid dispersion was manually applied onto the specimen’s surface using a paintbrush. The samples were coded as DNMS/GNPs, Triton/GNPs, and PSS/GNPs. Finally, the samples were left under a hood for drying. To determine the surface quantity of GNPs, the sample mass was weighed before and after deposition, and then the percentage by weight (wt%) was calculated.

## 3. Results

### 3.1. Scanning Electron Microscopy

**Figure 3:**
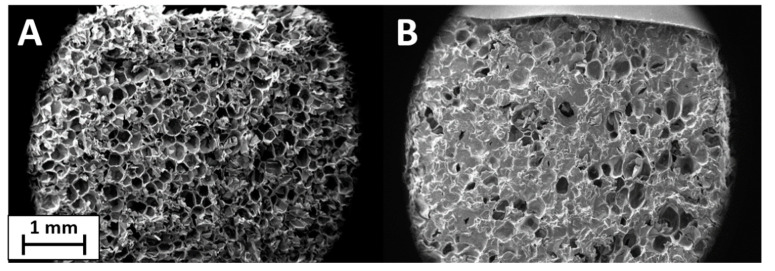
SEM images of untreated polyethylene terephthalate (PET) foam (**A**) and PET foam treated with PSS/GNPs coating (**B**).

SEM analysis was used to investigate the uniformity and thickness of the coating on the surface of the samples. The coatings were easily transferred to untreated PET foam with a closed-cell structure. The surface, clearly visible under magnification in Figure 3A, is formed by pores of 300–400 µm created by the closed-cell walls. This structure allows the coating to remain on the surface without going deep inside the foam. Figure 3B reports the image of PET foam treated with PSS/GNPs coating. The initial structure remains intact and the coating is good but not optimal, with some discontinuity points due to the characteristics of the solution. The coating is incomplete and not homogeneous on the whole surface due to the viscosity of the solutions. The low viscosity of PSS/GNPs and DNMS/GNPs causes the filling of the pores but not their walls, while the high viscosity of Triton/GNPs prevents the spreading by paintbrush of the homogeneous coating onto the surface. The coating thickness was about 150–200 µm, observed on SEM images from the section of the specimen (Supplementary materials Figure S1). SEM images of coatings from solutions of DNMS/GNPs and Triton/GNPs can be found in Supplementary materials Figure S2.

### 3.2. Flammability Tests

Horizontal and vertical flammability tests were performed on coated samples with the three solutions of GNPs prepared using different dispersants.

Horizontal tests were performed in order to calculate the burning rate (BR) of the samples. The untreated PET foam sample ignited after application of the first flame, when the flame was removed, it continued to burn until reaching the clamps, with a BR of 31 ± 1 mm/min. All coated samples were able to interrupt the spreading of flames after the two applications of 10 s. Furthermore, even after the third flame application of 30 s, the flame was interrupted before reaching the metallic clamp. The BRs were 48 ± 1 mm/min calculated for samples coated with DNMS solution, 18 ± 1 mm/min for Triton formulations, and 9 ± 1 mm/min for PSS-coated samples. The residues after testing were very significant: 93 ± 3, 63 ± 9, and 90 ± 2 wt%, for samples coated with DNMS, Triton, and PSS solutions, respectively.

Vertical testing for untreated PET foam leads to complete burning of all specimens during the first flame application. The flames spread easily along the sample, burning it completely (the final residue about 0 wt%) and dropping off molten material, which ignites the cotton placed under the specimen. The behavior of the Triton/GNPs sample is very similar to neat PET foam and was unable to pass the test. Instead, DNMS/GNPs and PSS/GNPs samples were capable of self-extinguishing behavior after the two flame applications. The coatings were able to flame out the sample, avoiding melt dripping and cotton ignition. The DNMS/GNPs sample passed the test with a V-2 ranking, and PSS/GNPs sample with a V-0 ranking due to the shorter afterflame times.

Regarding flammability tests, the PSS/GNPs solution was identified as the better surface treatment for fire-retardant coating. This formulation was able to extinguish the flames during horizontal tests even when the fire was forced, maintaining 90% of weight, a burning rate of 9 mm/min, and passing UL-94 in the vertical configuration with a V-0 ranking.

### 3.3. Flame Penetration Tests

**Figure 4:**
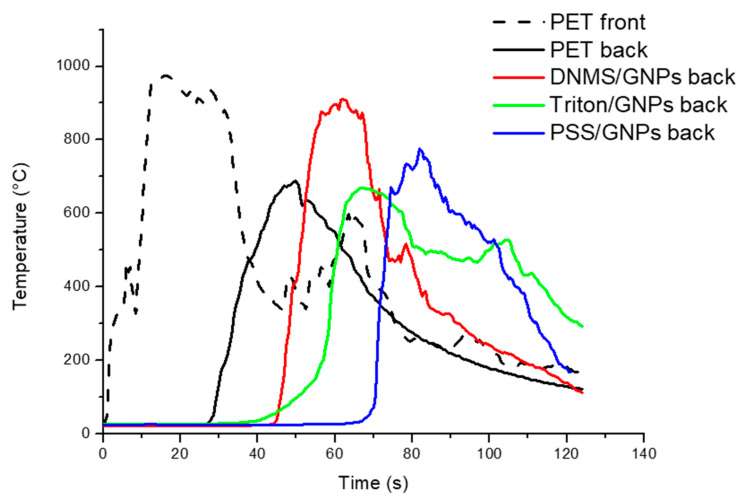
Flame penetration curves of samples with an exposed side covered by GNPs coating with solutions of DNMS, Triton, and PSS dispersants.

**Figure 5:**
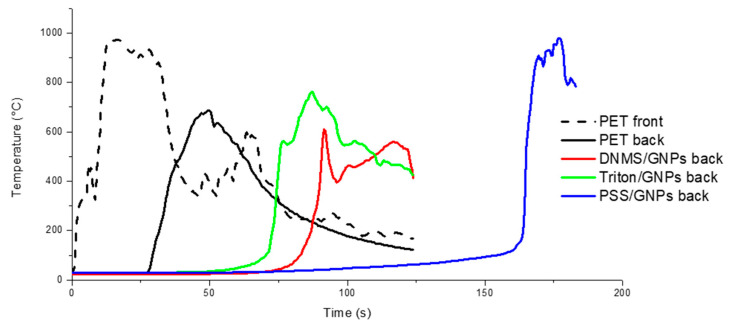
The flame penetration curves of samples with both sides covered by GNPs coating of solutions of DNMS, Triton, and PSS dispersants.

The flame penetration test was chosen to study the foam’ resistance to a direct flame, evaluating the flame penetration time and temperature on both sides of the specimen. Firstly, the samples were prepared with a coating covering one or both sides of each specimen. Temperatures, recorded using thermocouples on exposed (front) and unexposed sides (back), are reported in Figure 4 and Figure 5, with one side coated and both sides coated, respectively. Flame penetration times for different samples are listed in Table 1 and Table 2. Untreated PET foam ignited after seconds, involving temperatures that increased very quickly during the first 10 s until 1000 °C and reaching the unexposed face in 30 s. All the coated specimens were able to delay the ignition and complete penetration of the flame compared to neat PET foam, but there was more coating on both faces. Between them, PSS/GNPs specimens showed better performance: the temperatures remained under 400 °C on the exposed face for 60 s. On the back side, an increase in temperature was detected just after 70 s, when the flame was spreading across the entire sample.

When both faces were coated, the time of complete penetration of the sample was greatly extended for all the dispersant systems, reaching 165 s for PSS. By increasing the concentration of GNPs coating on both sides at 5.3 wt% from the PSS/GNPs solution, a huge extension of the full flame penetration time (210 s) was recorded. Oppositely, decreasing the concentration of GNPs to 1.8 wt% moved this parameter down to 130 s, which is still higher than the time of flame penetration of DMNS and Triton coatings at 3.8 wt% (85 and 70 s, respectively).

### 3.4. Cone Calorimetry Tests

**Figure 6:**
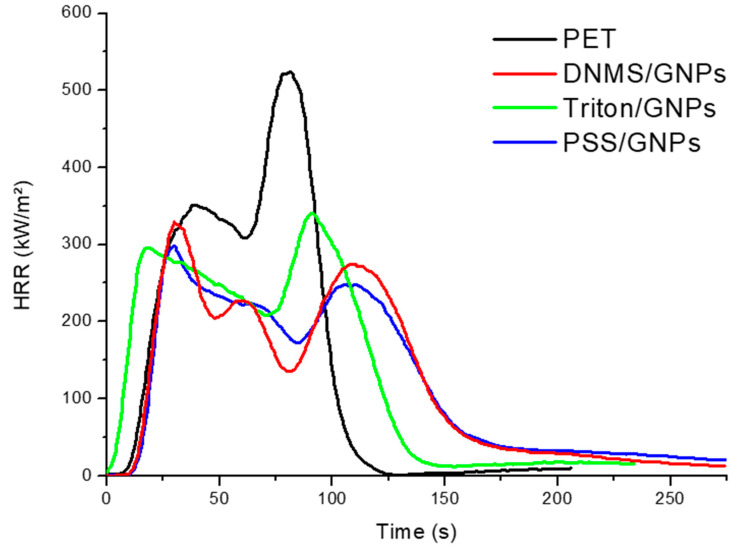
Heat release rate (HRR) vs. time curves for neat PET and coated sample with 2.5 wt% GNPs.

Combustion tests were first performed on the three different coatings with the same concentration of 2.5 wt% GNPs in order to evaluate the heat release rate (HRR), total heat release (THR), and times to ignition (TTI) of the specimens. Numerical results are reported in Table 3 and curves in Figure 6. All three coatings show enhanced fire-retardant properties compared to untreated PET foam. The HRR curves of neat PET foam exhibited two HRR peaks, with the second being higher than the first (603 and 370 kW m^−2^, respectively). The coated specimens presented a decrease in both peaks, especially the second, which was similar to the first and delayed in time with respect to neat PET. Furthermore, DNMS/GNPs and PSS/GNPs solutions permitted us to create coatings that are more resistant in terms of TTI, which delayed the fire by seconds. Further, in this case, the PSS/GNPs coating showed more improvements than other coatings. The pHRR was 303 kW m^−2^ (50% less than PET foam), while the THR and TTI remained similar to that of neat PET foam (THR = 32 ± 2 MJ m^−2^).

**Figure 7:**
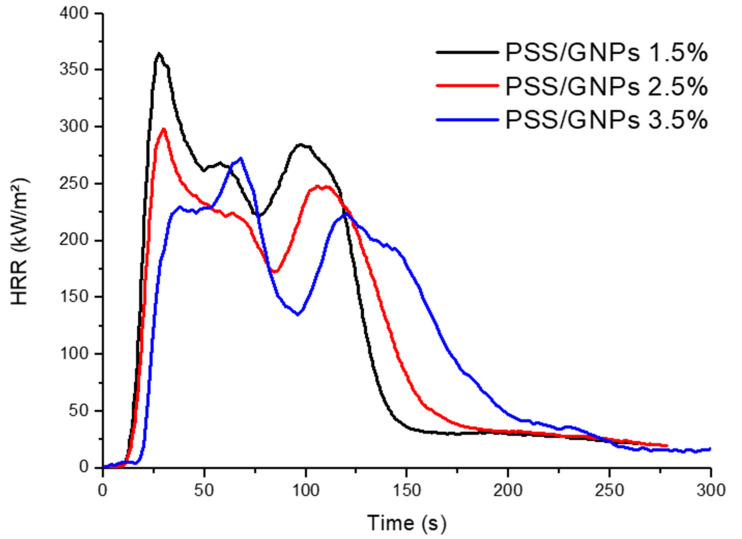
HRR vs. time curves for coated samples with different percentages of PSS/GNPs coating.

Once the solution with better fire performance was selected, specimens were prepared by changing the percentage of surface GNPs in order to evaluate their efficiency (Table 3 and Figure 7). Increasing the percentage of GNPs from 2.5 to 3.5 wt%, the pHRR decreased from 50% to 58% less than neat PET foam. Furthermore, TTI was still delayed to 28 s while THR remained almost the same. Coating with a lower concentration of GNPs of 1.5 wt%, however, decreased the pHRR of neat PET foam by 39%, and yielded a TTI of 21 s. Therefore, the flame retardant’s efficiency was closely related (with linear correlation) to the amount of coating deposition achieved.

### 3.5. Tests after Ageing Treatment

**Figure 8:**
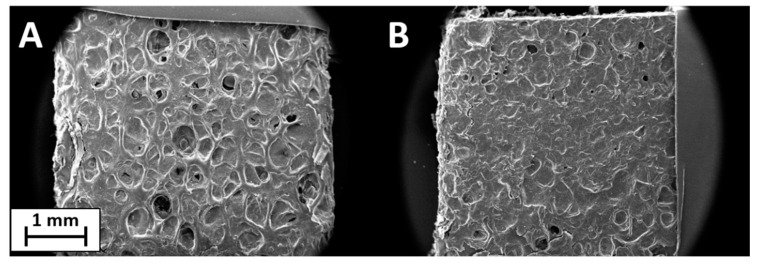
SEM images of PSS/GNPs coating after ageing treatment for 48 h (**A**) and 115 h (**B**).

PSS/GNPs coating showed the best results in previous tests, so the specimens were analyzed via ageing treatment in an oven at 160 °C for 48 h or 115 h. SEM images confirmed the effect of heat treatment, revealing a change in surface structure (Figure 8) compared to unaged specimens (Figure 3B). The surface structure of the heat-treated specimen was smooth and the walls of the pores were covered by the coating of GNPs. By extending the treatment time from 48 to 115 h, the surface evolved, becoming progressively flatter. The effect of heat treatment on flame-retardant properties was investigated using flame penetration and cone calorimeter tests.

During flame penetration tests on both heat-treated samples, the flame was not able to penetrate the specimens even after 400 s of flame application. Furthermore, temperatures of the exposed face remained constant below 300 °C, while the back face was quite stable between 25 and 35 °C.

**Figure 9:**
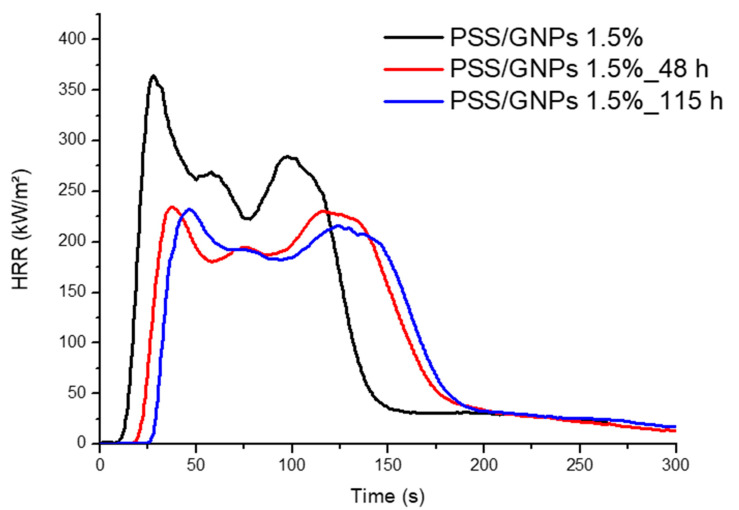
HRR vs. time curves for samples with 1.5 wt% of PSS/GNPs not aged and aged for 48 h and 115 h.

Cone calorimeter tests were performed on specimens with 1.5 wt% coating with PSS/GNPs solution and treated in an oven for 48 and 115 h at 160 °C (results in Table 3 and curves in Figure 9). An ageing treatment of 48 h was able to reach results similar to the untreated coated foam with 3.5 wt%: TTI of 29 s and reduction of neat PET foam by 58%. Samples were kept in an oven for 115 h and formed a superficial protection coating capable of resisting more exposure time to fire (TTI = 35 s) with a lower HRR (pHRR = 239 kW m^−2^, −60%).

## 4. Discussion

The application of GNPs coatings on PET foam showed excellent performance during flammability, flame penetration, and combustion tests. These improvements were related to new characteristics given to the foam by the coatings. PSS was identified as the better dispersant due to its viscosity and great affinity with GNPs. Indeed, the dispersant promoted the alignment of the nanoplatelets with an orientation parallel to the surface, creating a thermal barrier that limits the heat and gas exchange between condensed and gaseous phases [7,24,25].

Although GNPs are not a common flame retardant, in this study, we developed and optimized a novel method that is competitive with most frequently used additives. For example, Bethke et al. [26] studied and compared different commercial types of flame retardants containing halogen (HFR), phosphate (DOP), phosphinates (DEPZn), and phosphonates (DOP, PSMP), incorporated on PET foams via a reactive foam extrusion process. They were able to obtain specimens with a maximum decrease in pHRR equal to 31% for 2 wt% of PSMP using a cone calorimetry test. Carosio et al. showed, for the first time, an LbL deposition treatment on closed-cell polyethylene terephthalate (PET) foam with films of ammonium polyphosphate (APP) and DNA for flame-retardant application. It has been demonstrated that the performance of APP coatings is able to prevent melt dripping during flammability tests and reduces the pHRR by 25% during cone calorimetry tests [13].

**Figure 10:**
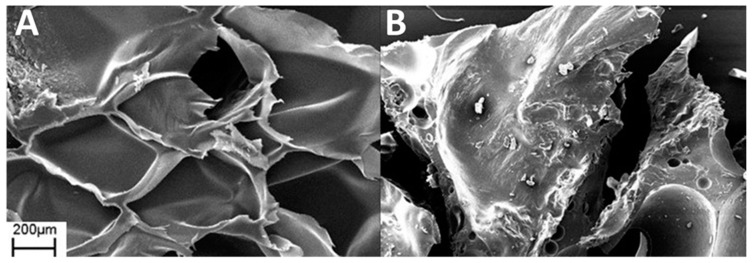
SEM images of PSS/GNPs coating (**A**) and PSS/GNPs coating after the vertical burning test (**B**).

**Figure 11:**
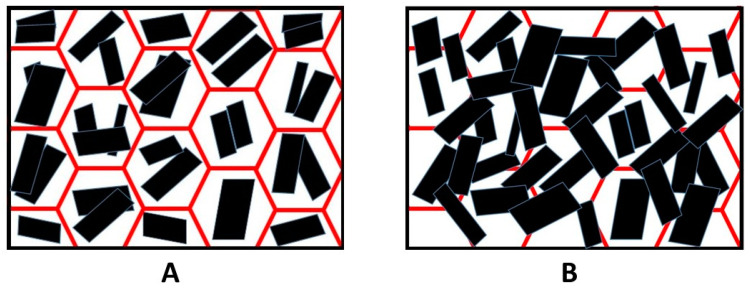
The scheme of surface of coated sample (**A**) and heat-treated coated sample (**B**).

The SEM analysis of the coated sample after the flammability test showed a continuous layer of GNPs and the walls of the pores were no longer distinguishable (Figure 10B) compared to the foam coated as prepared in Figure 10A. In Figure 11 is shown a scheme of that mechanism. Observing the surface uniformity, it was chosen to replicate the surface morphology with ageing treatments for the coated specimens.

The effect of the heat leads the evaporation of water, and the coating is continuously distributed on the surface by the dispersant. The heat treatment produces a surface structure (Figure 8) very similar to the burnt specimen observed in SEM images in Figure 10B. The result of the heat treatment was more complete coverage of the surface and walls covered by GNPs, which provided a more effective barrier. Flame penetration tests emphasized the excellent flame resistance properties of heat-treated specimens, which were capable of resisting the flame. Compared to literature, Carosio et al. [27] reported a method for preparing flame-resistant polyurethane foams via layer-by-layer assembly of graphene oxide nanoplatelets. The 6-bilayer (BL) coatings were capable of withstanding the penetration of an impinging flame torch (T surface ≈ 950 °C), insulating the unexposed side of the sample where the temperature remained ≤100 °C for the duration of the test.

## 5. Conclusions

Coated PET foams were successfully prepared from liquid solutions of GNPs and dispersant. The foam was treated with a simple, quick method for the manual application of the solution, capable of creating a stable coating on the foam’s surface. Different dispersants (PSS, Triton, and DMNS) were used in the solutions of liquid GNPs, and their efficiency with respect to flame-retardant properties was evaluated.

SEM images show how the foam’s structure is intact after application of the solution, and the coating remains on the surface. The distribution is good, but some points of discontinuity are visible, probably related to the viscosity of the solution.

Horizontal and vertical flammability tests show the significant effects of the coating on flame-retardant properties, especially with PSS/GNPs specimens. During the horizontal configuration test, these specimens were capable of self-extinguishing the flame with a very low burning rate and residue of about 90%. The vertical test passed with a V-0 ranking, avoiding the typical dripping of neat PET foams.

Flame penetration tests show the quick ignition of untreated PET foam involving temperatures of up to ~1000 °C in the first 10 s, and reaching the unexposed face in 30 s. All the coatings were able to delay the ignition and the complete penetration of the flame more effectively when both faces were coated instead of just one face. Among the three dispersants, the best performances were reached by coating with PSS/GNPs solutions: the specimen with the highest concentration of GNPs (5.3 wt%) reached complete penetration after 210 s of flame application. The strict relationship between time to penetration and the quantity of GNPs deposited was then demonstrated, because the times decreased when testing specimens with 1.8% and 3.8% GNPs (130 s and 165 s, respectively).

The results from cone calorimeter tests also show more improvements in the fire retardancy of the PSS/GNPs coating than other coatings. The highest-loaded PSS/GNPs specimen (3.5 wt%) has an HRR value 68% lower than untreated PET foam and a delay in TTI from 19 s to 28 s.

Specimens with PSS/GNPs coating were treated in an oven, providing an ageing effect on the surface. This procedure demonstrated huge benefits in flame-retardant properties during flame penetration and cone calorimeter tests. During the flame penetration test, specimens resisted the flame for more than 400 s. For a PSS/GNPs solution coating of just 1.5 wt%, HRR was 61% that of PET foam after an ageing treatment of 115 h at 160 °C. These excellent results were related to the conformation of the sample surface, which was observed via SEM. After heat treatment, the shape changed its original structure and the coating was continuously distributed without points of discontinuity.

## Figures and Tables

**Table 1:** Flame penetration times for PET foam and coated samples with one face treated.

Materials	Coating (wt%)	Time of Flame Penetration (s)
PET foam	-	30
DNMS/GNPs	3.0	50
Triton/GNPs	3.0	55
PSS/GNPs	3.0	70

**Table 2:** Flame penetration times for PET foam and coated samples with both faces treated.

Materials	Coating (wt%)	Time of Flame Penetration (s)
PET foam	-	30
DNMS/GNPs	3.8	85
Triton/GNPs	3.8	70
PSS/GNPs	3.8	165
PSS/GNPs	1.8	130
PSS/GNPs	5.3	210

**Table 3:** Cone calorimeter results for the tested materials at 35 kW m^−2^.

Materials	Coating (wt%)	TTI (s)	pHRR (kW m^−2^)	pHRR Reduction (%)
PET foam	-	19 ± 4	603 ± 84	-
DNMS/GNPs	2.5	23 ± 4	392 ± 18	35
Triton/GNPs	2.5	11 ± 2	343 ± 19	43
PSS/GNPs	2.5	22 ± 3	303 ± 12	50
PSS/GNPs	1.5	21 ± 2	371 ± 12	39
PSS/GNPs	3.5	28 ± 3	255 ± 14	58
PSS/GNPs (ageing 48 h)	1.5	29 ± 3	252 ± 4	58
PSS/GNPs (ageing 115 h)	1.5	35 ± 1	239 ± 10	60

## Supplementary Materials

The following are available online at https://www.mdpi.com/2073-4360/13/4/501/s1, Figure S1: SEM images of section fracture of PET foam coated with PSS/GNPs from different angles. Figure S2: SEM images of PET foam treated with DNMS/GNPs coating (A) and with Triton/GNPs coating (B).

## References

[1] Eaves D. (2004). Handbook of Polymer Foams.

[2] Lee L.J., Zeng C., Cao X., Han X., Shen J., Xu G. (2005). Polymer nanocomposite foams. Compos. Sci. Technol..

[3] Laoutid F., Bonnaud L., Alexandre M., Lopez-Cuesta J.M., Dubois P. (2009). New prospects in flame retardant polymer materials: From fundamentals to nanocomposites. Mater. Sci. Eng. R.

[4] Malucelli G., Carosio F., Alongi J., Fina A., Frache A., Camino G. (2014). Materials engineering for surface-confined flame retardancy. Mater. Sci. Eng. R.

[5] Paul D.R., Robeson L.M. (2008). Polymer nanotechnology: Nanocomposites. Polymer.

[6] Potts J.R., Dreyer D.R., Bielawski C.W., Ruoff R.S. (2011). Graphene-based polymer nanocomposites. Polymer.

[7] Trusiano G., Matta S., Bianchi M., Rizzi L.G., Frache A. (2019). Evaluation of Nanocomposites Containing Graphene Nanoplatelets: Mechanical Properties and Combustion Behavior. Polym. Eng. Sci..

[8] Lewin M., Pearce E.M., Levon K., Mey-Marom A., Zammarano A., Wilkie C.A., Jang B.N. (2006). Nanocomposites at elevated temperatures: Migration and structural changes. Polym. Adv. Technol..

[9] Lewin M. (2006). Reflections on migration of clay and structural changes in nanocomposites. Polym. Adv. Technol..

[10] Pastore H.O., Frache A., Boccaleri E., Marchese L., Camino G. (2004). Heat Induced Structure Modifications in Polymer-Layered Silicate Nanocomposites. Macromol. Mater. Eng..

[11] Colonna S., Cuttica F., Frache A. (2004). Aging of EVA/organically modified clay: Effect on dispersion, distribution and combustion behavior. Polym. Degrad. Stab..

[12] Decher G., Hong J.D. (1991). Buildup of ultrathin multilayer films by a self-assembly process, 1 consecutive adsorption of anionic and cationic bipolar amphiphiles on charged surfaces. Makromol. Chem. Macromol. Symp..

[13] Carosio F., Cuttica F., Di Blasio A., Alongi J., Malucelli G. (2015). Layer by layer assembly of flame retardant thin films on closed cell PET foams: Efficiency of ammonium polyphosphate versus DNA. Polym. Degrad. Stab..

[14] Chiang C.-L., Wang F.-Y., Ma C.-C.M., Chang H.R. (2002). Flame retardance and thermal degradation of new epoxy containing silicon and phosphorous hybrid ceramers prepared by the sol-gel method. Polym. Degrad. Stab..

[15] Liu Y.-L., Chou C.-I. (2005). The effect of silicon sources on the mechanism of phosphorus-silicon synergism of flame retardation of epoxy resins. Polym. Degrad. Stab..

[16] Chiang C.-L., Ma C.-C.M., Wu D.-L., Kuan H.-C. (2003). Preparation, Characterization, and Properties of Novolac-Type Phenolic/SiO2Hybrid Organic-Inorganic Nanocomposite Materials by Sol-Gel Method. J. Polym. Sci. Part A Polym. Chem..

[17] Messori M., Toselli M., Pilati F., Fabbri E., Fabbri P., Busoli S., Pasquali L., Nannarone S. (2003). Flame retarding poly (methyl methacrylate) with nanostructured organic-inorganic hybrids coatings. Polymer.

[18] Ji Q., Wang X., Zhang Y., Kong Q., Xia Y. (2009). Characterization of Poly (ethylene terephthalate)/SiO2 nanocomposites prepared by Sol-Gel method. Compos. Part A Appl. Sci. Manuf..

[19] Alongi J., Frache A., Malucelli G., Camino G., Kilinc F.S. (2013). Multi-component flame resistant coating techniques for textiles. Handbook of Fire Resistant Textiles.

[20] Alongi J., Ciobanu M., Tata J., Carosio F., Malucelli G. (2011). Thermal Stability and Flame Retardancy of Polyester, Cotton, and Relative Blend Textile Fabrics Subjected to Sol-Gel Treatments. J. Appl. Polym. Sci..

[21] Bourbigot S., Bachelet P., Samyn F., Jimenez M., Duquesne S. (2013). Intumescence as method for providing fire resistance to structural composites: Application to poly (ethylene terephtalate) foam sandwich-structured composite. Compos. Interfaces.

[22] Cesareo G., Parrini M.R., Rizzi L.G. (2014). Concentrated Water Dispersion of Graphene and Method for the Preparation Thereof.

[23] Bonetti L., Fiorati A., Serafini A., Masotti G., Tana F., D’Agostino A., Draghi L., Altomare L., Chiesa R., Farè S. (2021). Graphene nanoplatelets composite membranes for thermal comfort enhancement in performance textiles. J. Appl. Polym. Sci..

[24] Kalantari B., Mojtahedi M.R.M., Sharif F., Rahbar R.S. (2015). Flow-induced crystallization of polypropylene in the presence of graphene nanoplatelets and relevant mechanical properties in nanocompsoite fibres. Compos. Part A Appl. Sci. Manuf..

[25] Caradonna A., Colucci G., Giorcelli M., Frache A., Badini C. (2017). Thermal behavior of thermoplastic polymer nanocomposites containing graphene nanoplatelets. J. Appl. Polym. Sci..

[26] Bethke C., Goedderz D., Weber L., Standau T., Döring M., Altstädt V. (2020). Improving the flame-retardant property of bottle-grade PETfoam made by reactive foam extrusion. J. Appl. Polym. Sci..

[27] Carosio F., Maddalena L., Gomez J., Saracco G., Fina A. (2018). Graphene Oxide Exoskeleton to Produce Self-Extinguishing, Nonignitable, and Flame Resistant Flexible Foams: A Mechanically Tough Alternative to Inorganic Aerogels. Adv. Mater. Interfaces.

