# Supplementary material for: PET Foams Surface Treated with Graphene Nanoplatelets: Evaluation of Thermal Resistance and Flame Retardancy
Source: Polymers (Basel). 2021 Feb 6;13(4):501. doi: 10.3390/polym13040501 (PMC7914555; doi:10.3390/polym13040501)
Supplement: Supplementary file 1 [file polymers-13-00501-s001.pdf]

PET foams surface treated with graphene nanoplatelets: evaluation of thermal resistance and flame retardancy

Samuele Matta, Laura Giorgia Rizzi and Alberto Frache

Supplementary materials

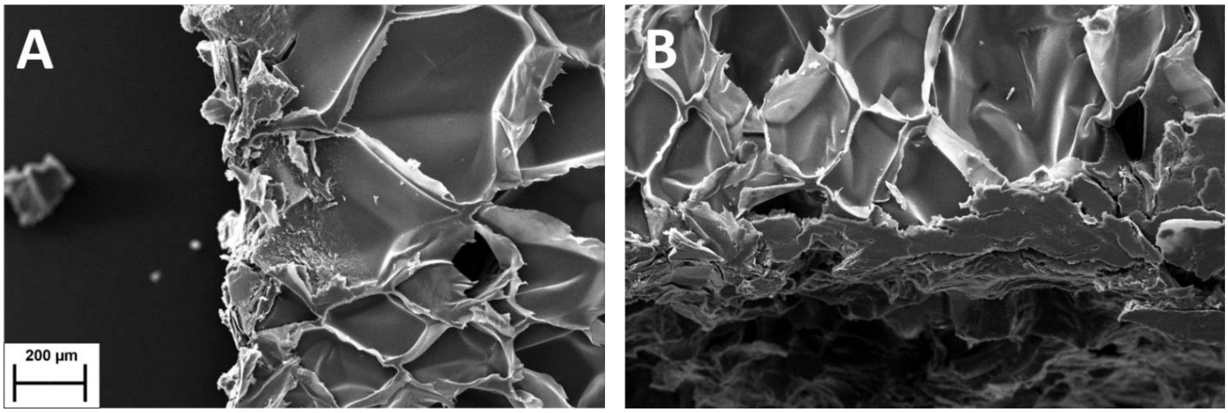

Figure S1: SEM images of section fracture of PET foam coated with PSS/GNPs from different angles.

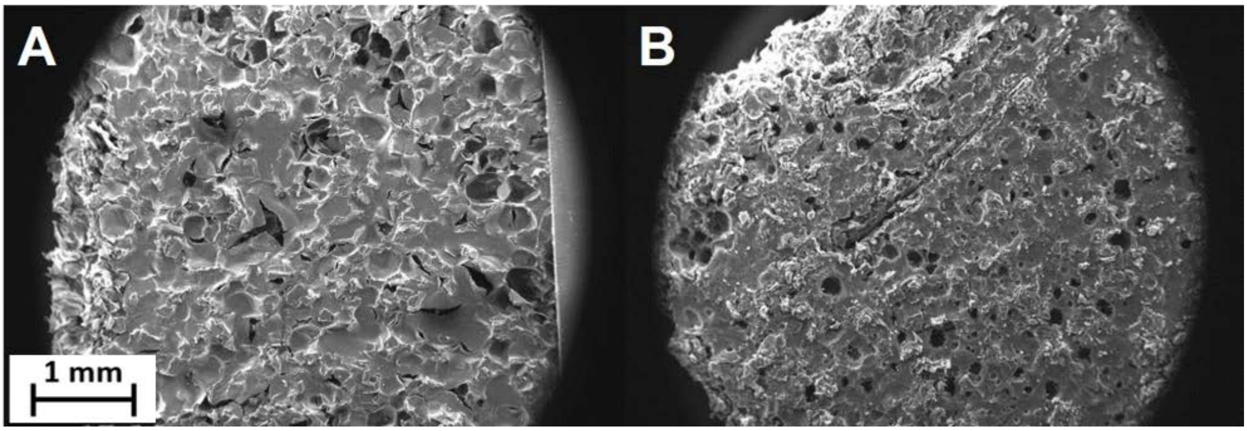

Figure S2: SEM images of PET foam treated with DNMS/GNPs coating (A) and with Triton/GNPs coating (B).
